# Zika virus evolution on the edges of the Pacific ocean

**DOI:** 10.1038/emi.2017.102

**Published:** 2017-12-13

**Authors:** Myrielle Dupont-Rouzeyrol, Laure Diancourt, Elodie Calvez, Mathias Vandenbogaert, Olivia O'Connor, Anita Teissier, Morgane Pol, Maite Aubry, Oumar Faye, Douglas Tou, Van-Mai Cao-Lormeau, Valérie Caro

**Affiliations:** 1 Institut Pasteur de Nouvelle-Calédonie, Réseau International des Institut Pasteur, URE Dengue et Arboviroses, 98 845 Noumea, New Caledonia, France; 2 Institut Pasteur, Environment and Infectious Risks Unit, Laboratory for Urgent Response to Biological Threats, 75 724 Paris, France; 3 Institut Louis Malardé, 98 713 Papeete, French Polynesia, France; 4 Institut Pasteur de Dakar, 16 000 Dakar, Senegal; 5 Rarotonga Hospital Laboratory, TE MARAE ORA—Ministry of Health, Rarotonga, Cook Islands

**Dear Editor,**

Over the past decade, arthropod-borne viruses (arboviruses) including dengue virus (DENV), chikungunya virus (CHIKV) and Zika virus (ZIKAV), have demonstrated their potential to pose major global public health problems. Several outbreaks caused by these viruses recently occurred in the Pacific region, probably resulting from multiple factors:^1^ the presence of competent mosquito vectors; environmental and demographical conditions favourable to mosquito proliferation and disease transmission; and the increasing volume of travel between continental tropical areas where arboviruses are endemic and the Pacific, and between Pacific Island Countries and Territories (PICTs). In 2013, ZIKAV emerged in French Polynesia and subsequently spread to other PICTs.^1^ In 2015, ZIKAV appeared in Brazil and several other Latin American countries where it was associated with a marked increase in the number of cases of congenital abnormalities, including microcephaly, and neurological disorders.^2, 3, 4^ Phylogenetic analysis classified ZIKAV into two major genetic lineages, African and Asian, with the Asian lineage responsible for the current global expansion of ZIKAV.^2^ To date, except for French Polynesia, there are little data on ZIKAV Pacific strains.^5, 6^ In our study, by adding 13 new full ZIKAV genome sequences, isolated from different places in the Pacific region and at different periods of time, along with other published genomes, we provide for the first time a map of the whole ZIKAV Pacific sublineage, from the Western to the Eastern edges of the Pacific ocean.

The 13 ZIKAV strains used in this study were isolated from human patients in French Polynesia, New Caledonia, Cook Islands and Vanuatu between 2013 and 2015 (Supplementary Table S1). Full ZIKAV genomes were obtained by in-house high-throughput sequencing with the Ion Personal Genome Machine (PGM) sequencer (Thermofisher Scientific/Life Technologies, Carlsbad, CA, USA). Briefly, viral RNA was extracted from serum, saliva swab or cell culture supernatant (obtained by inoculating serum or breast milk^7^ on Vero cells) with QIAmp Viral RNA Mini kit according to the manufacturer’s recommendations (Qiagen, Hilden, Germany; Supplementary Table S1). Treated with Turbo Dnase Enzyme (Invitrogen/Life Technologies), viral RNA was purified using Agencourt RNAClean XP beads (Beckman Coulter, Beverly, MA, USA). cDNA synthesis was performed with SuperScript VILO cDNA synthesis kit according to the manufacturer’s recommendations (Thermofisher Scientific/Life Technologies). Libraries were prepared with Ion AmpliSeq Library 2.0 kit (Life Technologies, Carlsbad, CA, USA). We designed two custom and specific ZIKAV panels (www.ampliseq.com; Ion AmpliSeq Designer), giving 39 amplicons covering all the genome of ZIKAV. Each library was barcoded using the Ion Xpress Barcode Adapter 17-32 kit (Life Technologies) and quantified with Ion Library Taqman quantification kit (Thermofisher Scientific/Life Technologies). Barcoded libraries were combined to a final concentration of 60 pM and loaded onto 316 (100 Mb output data) chips, using Ion Chef System (Life Technologies) and sequencing was carried out on the PGM System (Life Technologies). Data analysis was performed according to the sequencing depth provided by the genetic analyser. The genome for isolate Pf13/251013-18 was *de novo* assembled using CLC assembly cell (http://www.clcbio.com/products/clc-assembly-cell/) version 4 and standard parameters, yielding one contig (11 155 bp). Genomes from samples NC-14-2743, NC-14-7000 and CK-14-48/01 were assembled using a directed MIRA v4 assembly, using the Pf13/251013-18 as reference assembly. Finally, for the nine other samples, mapping of raw sequencing data was performed with Burrows Wheeler Alignment on Pf13/251013-18 assembly, from which a consensus was derived (using SAMTOOLS package, version 0.1.19, http://samtools.sourceforge.net/). The average assembled sequence length is 10 666.7 bp (range 10 454–11 155 bp). Average sequencing depth was around 4000 ×. Evolutionary rates were estimated using Bayesian Evolutionary Analysis by Sampling Trees (BEAST) v1.8.4 (https://tree.bio.ed.ac.uk/wiki/projects/beast/BEAST.html), on the 13 ZIKAV full genomes sequenced in this study and 72 additional sequences retrieved from GenBank (alignment available upon request). The SRD06 codon-partitioned model was applied using the general time reversible nucleotide substitution model with gamma distribution, a strict molecular clock with a continuous-time Markov chain prior and a Bayesian skyline coalescent tree prior with a piecewise-constant demographic model. Both mixing of individual chains and sufficient effective sample size was ensured by running each data set eight times for 100M generations, sampling every 10k generations. After discarding 10% burn-in for each run, consensus files for each data set were produced using LogCombiner and TreeAnnotator (BEAST packages). Consensus trees were viewed and validated using FigTree 1.3.1 (http://tree.bio.ed.ac.uk/software/figtree/).

The 13 new full ZIKAV genome sequences obtained with this work are belonging to the Asian lineage (Figure 1). All the 13 ZIKAV strains are harbouring several amino-acid substitutions, including 139N, 683E, 777M and 763M, which are specific to the ZIKAV strain initially isolated in French Polynesia (PF-251013-18; KX369547) and to all the ZIKAV Pacific strains genomes available as previously described.^5^

Phylogenetic analysis of ZIKAV full genomes from various locations confirms African and Asian as two distinct lineages (data not shown) with a last common ancestor around 1822 (95% highest posterior density interval (HPD), 1788 to 1838) and the emergence of Asian lineage around 1942 (95% HPD, from 1934 to 1950). Our analysis also suggests that the ancestor of the French Polynesian strains emerged roughly during the year 2012 (95% HPD, September 2011 to September 2012).

For phylogenetic analysis, we first constructed phylogenetic trees with all complete genomes (465 and 196) available in July 2017. However, for clarity and because topology of the tree was very comparable, the phylogenetic analysis presented in this study was reduced to the 13 ZIKAV full genomes sequenced and 72 additional sequences retrieved from GenBank (with a special focus on Pacific region sequences; Figure 1). This analysis supports that the ZIKAV strains that caused the outbreaks in the Pacific and in Latin America^6^ evolved into two distinct sublineages (supported by a posterior probability value of 0.99) that circulated independently within the two areas from 2015 (Figure 1). The tree highlights the diversity over time commonly observed with such arbovirus, with a striking collocation of the strains according to the geographical origin of sampling. Interestingly the phylogenetic tree seems to reveal two distinct clusters within the Pacific sublineage supported by a posterior probability value of 0.93, although there are no striking amino-acid changes. A first cluster contains French Polynesian strains, including those that appear in the Latin American sublineage, and all strains from New Caledonia and Vanuatu. A second cluster contains other strains from French Polynesia and ZIKAV strains from Fiji, American Samoa and the Cook islands. These observations suggest that ZIKAV dissemination throughout the Pacific region followed at least two distinct pathways. One is probably resulting from the existence of close links and regular flight connections between French Polynesia, New Caledonia and Vanuatu. This is also corroborated by epidemiological data from New Caledonia health authorities reporting 30 ZIKAV imported infections from French Polynesia by the end of 2013, followed by the detection of the first autochthonous ZIKAV cases in January 2014.^8, 9^ The dissemination of arboviruses between French Polynesia, New Caledonia, and Vanuatu was already observed at several occasions by the past, notably for DENV-1 and DENV-4.^10, 11^ The second pathway of dissemination may have been driven by travel exchanges between French Polynesia and other PICTs; and between those PICTs.

Our study shows that whatever the island country in the Pacific, and whatever the year the ZIKAV strains were isolated (2013, 2014, 2015 and 2016), all viral strains cluster together with French Polynesia strains. The finding that all Pacific strains (except two French Polynesian strains) belong to the same Pacific sublineage supports that ZIKAV dissemination throughout the Pacific region originated in French Polynesia without any further importation of ZIKAV from continental areas. This observation contrasts with data on the other arboviruses (CHIKV, DENV-1 and DENV-3) that circulated concomitantly to ZIKAV in the Pacific region^12, 13, 14^ and to what happened in the Americas with multiple introductions of ZIKAV from the Caribbean region and Central/South America in the United States.^15^

The Pacific region was at the beginning of the ZIKAV globalization. Our results suggest that the ZIKAV Pacific sublineage is certainly due to a single introduction in French Polynesia and subsequent spread to other PICTs. This work highlights the need for a better understanding of the patterns of introduction and dissemination of arboviruses in the Pacific region as it could help to mitigate future outbreaks.

## Figures and Tables

**Figure 1:**
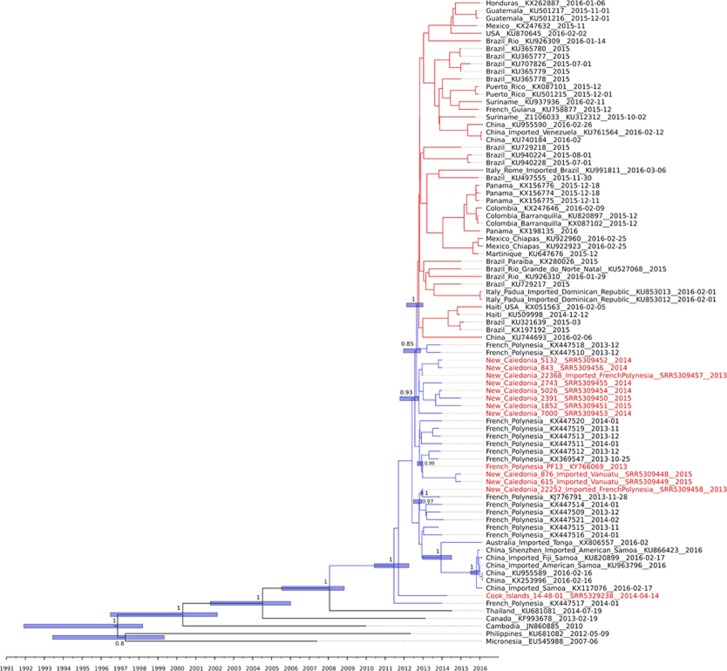
Zika virus phylogeny of Asian/Pacific and Latin American virus isolates. For clarity, the tree displays all Pacific virus branches in blue and all Latin American virus branches in red. The 13 virus sequences obtained in this study are colored in red text. Blue bars represent highest posterior density interval (HPD 95%). Because of the relatively recent emergence of a high number of Zika virus strains, the posterior probability support values are lower for Pacific lineages than in the phylogenetic tree that includes Malaysian and African lineages, giving stronger phylogenetic support (data not shown). Furthermore, to better interpret the phylogenetic analysis, the tree was produced by excluding the African and the Malaysian lineage by focusing on Pacific-representative sequences and by removing highly similar sequences. The sequences of the Pacific isolates obtained in this study have been submitted to GenBank in the form of Sequence Read Archives, under accession no. SRR5329238 and SRR5309448 to SRR5309458, and for strain Pf13/251013-18 as Genbank accession number KY766069.
