# Supplementary material for: Zika virus evolution on the edges of the Pacific ocean
Source: Emerg Microbes Infect. 2017 Dec 13;6(12):e111–. doi: 10.1038/emi.2017.102 (PMC5750458; doi:10.1038/emi.2017.102)
Supplement: Supplementary Table S1 [file emi2017102x1.docx]

**Supplementary Table S1. Zika virus strains analyzed in the study**

| **Isolate Genbank Identification** | **Sample origin** | **Geographical origin** | **Isolation date** | **Accession Number** | **Coverage relative to Pf13/251013-18 (%)** |
| --- | --- | --- | --- | --- | --- |
| NC(imp FP)-13-22252 | Cell supernatant | New Caledonia (imported French Polynesia) | 2013 | SRR5309458 | 0.938682 |
| NC(imp FP)-13-22368 | Cell supernatant | New Caledonia (imported French Polynesia) | 2013 | SRR5309457 | 0.921381 |
| NC-14-843 | Cell supernatant | New Caledonia | 2014 | SRR5309456 | 0.938861 |
| NC-14-2743 | Cell supernatant | New Caledonia | 2014 | SRR5309455 | 0.991215 |
| NC-14-5026 | Cell supernatant | New Caledonia | 2014 | SRR5309454 | 0.938861 |
| NC-14-5132 | Cell supernatant | New Caledonia | 2014 | SRR5309452 | 0.938682 |
| NC-15-1852 | Cell supernatant | New Caledonia | 2015 | SRR5309451 | 0.937517 |
| NC-15-2391 | Cell supernatant | New Caledonia | 2015 | SRR5309450 | 0.939220 |
| NC-14-7000 | Cell supernatant | New Caledonia | 2014 | SRR5309453 | 0.987808 |
| NC(imp VU)-15-615 | Serum | New Caledonia (imported Vanuatu) | 2015 | SRR5309449 | 0.937517 |
| NC(imp VU)-15-876 | Serum | New Caledonia (imported Vanuatu) | 2015 | SRR5309448 | 0.937517 |
| CK-14-48/01 | Saliva swab | Cook Islands | 2014 | SRR5329238 | 0.985119 |
| Pf13/251013-18 | Cell supernatant | French Polynesia | 2013 | KY766069 | 1.0 |

Cell supernatant was obtained by inoculating serum or breast-milk (NC-15-2391) on mammalian VERO cells. The sequences of the Pacific isolates obtained in this study have been submitted to GenBank in the form of Sequence Read Archives, under accession no. SRR5329238 and SRR5309448 to SRR5309458, and for strain Pf13/251013-18 as Genbank accession number KY766069. Genome coverage values relative to Isolate Pf13/251013-18 are mentioned.
